# Preliminary effectiveness of a food pantry-led community-supported agriculture program in the USA: a quasi-experimental pilot study

**DOI:** 10.1017/S136898002610264X

**Published:** 2026-06-02

**Authors:** Ruyu Liu, Daniel Harwood, Ora Kemp, Alyson Rosenthal, Roger Figueroa

**Affiliations:** 1 Division of Nutritional Sciences, College of Human Ecology, Cornell University, Ithaca, NY 14853, USA; 2 New York Common Pantry, New York, NY 10029, USA; 3 West Side Campaign Against Hunger, New York, NY 10024, USA

**Keywords:** Skin carotenoids, Food security, Emergency food assistance, Fresh produce access, Food access

## Abstract

**Objective:**

To assess the preliminary effectiveness of a food pantry-led, subsidised community-supported agriculture (CSA) program.

**Design:**

A quasi-experimental observational pilot study with 3-month follow-up. ‘Farm Share’ the subsidised CSA provided biweekly bags of approximately 25 pounds of fresh produce priced at $5 per bag. Control participants were recruited from a comparable food pantry without CSA. Outcomes included skin carotenoids, fruit and vegetable (FV) intake, diet quality and food security. Changes between groups were analysed using generalised estimating equations and mixed-effect regression models.

**Setting:**

New York City, NY, United States.

**Participants:**

Participants (Farm Share *n* 54, control *n* 69) were predominantly Hispanic/Latino, Black or African American, foreign-born, female and had a high school education or lower.

**Results:**

Retention rate was 65·0 % at follow-up (Farm Share *n* 33, control *n* 47). Compared with control, skin carotenoids increased (+12 points) in the Farm Share group, adjusted for smoking status, age, sex and birthplace (*P* = 0·005). Total healthy eating index (HEI) (–6·3 points) and saturated fats HEI (–1·1 point) declined in the Farm Share group (*P* = 0·031 and *P* = 0·027, respectively). Non-significant trends were observed for improvements in FV intake (*P* = 0·25) and food security (*P* = 0·16).

**Conclusions:**

Findings from this pilot study suggested Farm Share’s preliminary effectiveness and study feasibility in urban food pantry settings, supporting integrating subsidised CSA within food pantries to enhance fresh produce access among communities experiencing food insecurity. However, fully powered trials are needed to confirm these findings.

Suboptimal fruit and vegetable (FV) intake is a global public health issue, associated with higher risks of chronic diseases such as CVD, type 2 diabetes and stroke^(1)^. Despite the Dietary Guidelines for Americans recommending adults aged 19–59 years consume approximately five cups of FV daily, the average adult in the USA falls short of this target^(2)^. This gap is particularly pronounced among populations with lower socio-economic status (SES), such as food pantry clients^(3)^.

Emergency food assistance organisations, such as food pantries in the USA (commonly referred to as ‘food banks’ in European countries), are vital components of the charitable food systems that provide direct food assistance to individuals in need^(4)^. These organisations often reach vulnerable populations who may fall outside governmental assistance programs, with up to 60·9 % of pantry clients not receiving federal food benefits^(5)^. Additionally, the influence of food pantries on clients’ diets is substantial, as pantry-sourced foods can comprise up to 25% of clients’ total food intake^(6)^. This significant contribution to household food supply, combined with their broad community reach, positions food pantries as strategic settings for improving FV intake and food security among populations with lower SES.

Common food pantry interventions for improving dietary outcomes have included nutrition education, cooking demonstrations and disease-specific food provision^(3,7,8)^. Community-supported agriculture (CSA), where consumers pre-pay farmers for regular shares of seasonal produce throughout the growing season^(9)^, represents an innovative but understudied approach in food pantry settings. While CSA shows promise for improving FV intake, its traditional advance payment structure often creates barriers for populations with lower SES^(10,11)^. In response, subsidised CSA models emerged, offering cost-reduced weekly payment options.

Growing evidence supports subsidised CSA’s effectiveness in improving dietary behaviours and food security among low-SES populations^(12–18)^. For example, a large randomised controlled trial in the USA (the Farm Fresh Foods for Healthy Kids study, F3HK) examined 148 parent/caregiver–child dyads across twelve farm communities^(18)^. This study demonstrated significant improvements in caregiver FV intake, skin carotenoids, home FV availability, FV access and household food security, adding to the international evidence base for CSA’s potential impact on vulnerable populations^(18)^. While these findings are promising, existing research has primarily examined researcher-led subsidised CSA interventions outside of food pantry settings.

To address these gaps, this pilot study evaluated a community-led subsidised CSA program (‘Farm Share’) within a food pantry, examining both its preliminary effectiveness on key outcomes (food security, diet quality, FV intake and skin carotenoids) and the feasibility of the study approach.

## Methods

### Program overview

The Farm Share program was designed and implemented by a food pantry (‘CSA pantry’ hereafter) located in South Bronx and East Harlem of New York City (NYC) in the USA. East Harlem and South Bronx exhibit higher rates of poverty, food insecurity, governmental food assistance recipients, obesity, diabetes and hypertension compared with the city averages. The residents in these two neighbourhoods predominantly identify as Black or Hispanic^(19,20)^. The CSA pantry is one of the largest food pantries in NYC, offering a variety of services in addition to Farm Share, including biweekly pantry packages (shelf-stable foods and produce), hot meals, to-go lunches, monthly groceries for seniors, nutrition education workshops and cooking demonstrations. Social services were also provided, such as hygiene services and assistance to access public housing and social benefits. In fiscal year 2023 (when the study took place), the CSA pantry provided over 10 million meals and served 667 734 individuals, with approximately 31 185 volunteers hours. Most of the CSA pantry clients identified as Black, Hispanic/Latino and spoke Spanish.

The Farm Share program was subsidised by the Supplemental Nutrition Assistance Program Education (SNAP-Ed), the educational component of SNAP, the largest federal food assistance program in the USA. The Director of Nutrition and Nutrition Programs Manager at the pantry worked with a year-round regional farm food distributor to provide biweekly shares of approximately 25–30 pounds of fresh, seasonal produce (13–15 items in total) valued at $25–30. Participants paid $5 cash per box. Each box was accompanied by an educational flyer with nutrient information and instructions for cooking and storing the produce. All SNAP-eligible pantry members, based on self-report income information, were eligible to participate. Farm Share boxes were distributed at the three pantry locations in the East Harlem and South Bronx areas on Tuesday mornings every other week. Bilingual (i.e. English and Spanish) pantry nutritionists delivered biweekly virtual nutrition education and cooking demonstrations using Farm Share produce, open to all pantry clients regardless of Farm Share participation.

### Study design

This pilot study used an observational, quasi-experimental, 3-month pre-post design. The control pantry selection criteria included (1) serving a similar population in terms of age, race and ethnicity, (2) not currently having a CSA program or similar fresh FV programs and (3) not located in the South Bronx or East Harlem to decrease the likelihood of dependent observations. Initial recruitment efforts through NYC’s borough-wide food pantry directory yielded no responses despite email and phone outreach. As an alternative approach, the CSA pantry staff connected the research team with their ‘sister pantry’ in the Upper West Side, a borough that has higher median household income, lower rates of poverty, food insecurity, obesity and diabetes compared to NYC averages, with over 60 % of residents identifying as White^(21)^.

While no direct comparisons between pantries were available due to their different impact reporting metrics, multiple meetings and visits with research team confirmed that the control pantry met inclusion criteria. Although located in a neighbourhood with predominantly White populations, the control pantry served a largely Hispanic/Latino, Spanish-speaking clientele. Additionally, pantry internal zip code data showed that the majority of control pantry clients were not Upper West Side residents but came from neighbourhoods similar to those served by the CSA pantry.

In fiscal year 2023, the control pantry distributed 3·9 million pounds of food to over 80 000 individuals, with more than 2000 volunteers contributing 20 000 service hours. Members were eligible to pick up monthly pantry boxes containing both shelf-stable foods and produce, with each box including approximately five varieties of vegetables and three varieties of fruits, totaling about 16 pounds. The pantry also provided comprehensive social services, including assistance with SNAP enrollment, health insurance navigation and referrals for legal support and affordable housing.

Participants in the Farm Share group purchased Farm Share boxes up to twice a month while control participants did not. Both groups were free to receive regular pantry items and services as they normally would.

### Recruitment

A bilingual research assistant conducted recruitment on a rolling basis at both food pantries during operational hours from March to August 2023. Eligibility criteria included: (1) proficiency in English or Spanish, (2) age 18 or older, (3) receipt of food from either pantry at least once within the past year, (4) intent to remain in residence for at least 3 months, (5) access to a phone and (6) no prior receipt of CSA services from the study pantry. Eligible participants were provided with the informed consent form, and any questions were addressed before obtaining verbal consent.

### Baseline and follow-up data collection

After providing informed consent, participants completed a survey either on paper or via Qualtrics using their mobile devices, with the majority opting for the paper format. The survey included questions on demographic characteristics, food security and FV intake and required approximately 10 min to complete. Research assistants and pantry staff provided reading assistance when needed. Participants then underwent a brief skin carotenoid assessment lasting less than 5 min. Due to their time and cognitive demands, 24-h dietary recalls were offered as an optional component, with interested participants contacted within 1 week of enrollment for up to two phone-based, interviewer-administered recalls. Three months after enrollment, the RA contacted participants to schedule follow-up assessments at their respective pantries, which were identical to baseline data collection. At both time points, participants received $10 cash for completing the survey and skin carotenoid assessment and an additional $10 cash for each dietary recall completed.

### Outcome variables

FV intake was estimated using the abbreviated Dietary Screener Questionnaire, a ten-item assessment of FV intake^(22)^. Following National Cancer Institute scoring procedures, frequency responses were converted to daily equivalents (e.g. ‘1 time last month’ = 0·033 daily). Daily cup equivalents were then calculated for each FV item using age and sex-specific portion sizes, intake frequencies and established regression coefficients. A few participants (*n* 3 at baseline, *n* 1 at follow-up) had missing responses for at least one question. Two participants missed the question about fruit juice, one missed the question about other vegetables and one missed the questions about other potatoes, dried beans, other vegetables and tomato sauce. In the last case, these questions were consecutive and printed on one entire page, likely resulting from the failure to separate pages. Given the minimal missing data with no apparent patterns, complete data analysis was conducted.

Skin carotenoids were assessed using the Veggie Meter® (Longevity Link Corporation, 2015), a portable and non-invasive device using pressure-mediated reflection spectroscopy^(23)^. Scores range from 0 to 800, with higher values indicating higher carotenoid levels. The skin carotenoid scores have been used as a proxy for FV intake, showing strong correlations with serum carotenoids among non-Hispanic Black (*r* = 0·64) and Hispanic/Latino populations (*r* = 0·80)^(24)^. Self-reported weight, height, age and smoking status were collected prior to the assessment. The index finger of the non-dominant hand was cleaned with alcohol prep pads and placed in the device for scanning. The final score was the average score generated from the triplicate mode, which provides higher accuracy and reliability compared with manually averaging three single scans^(25)^.

Diet quality was assessed using the Healthy Eating Index (HEI), calculated from the diet recall data using the Nutrition Data System for Research software (version 2022, the Nutrition Coordinating Center, University of Minnesota, Minneapolis, MN)^(26)^. The dietary recall, a structured interview about foods, beverages and supplements consumed during the previous day, included detailed probes about brand names, cooking methods and portion sizes^(27)^. The HEI scores are based on thirteen key food groups in the Dietary Guidelines for Americans 2020–2025^(28)^. The sum of the thirteen component scores is the total HEI score, ranging from 0 to 100. A higher HEI score indicates better diet quality or closer adherence to the Dietary Guidelines for Americans 2020–2025.

Food security was measured using the Six-Item Short Form following the data analysis and interpretation procedures recommended by the U.S. Department of Agriculture^(29)^. Affirmative responses include ‘often’ or ‘sometimes’ on questions HH3 and HH4, ‘yes’ on AD1, AD2 and AD3, and 3 d or more on AD1a. Raw scores (sum of affirmative responses) indicate high or marginal food security (0–1), low food security (2–4) or very low food security (5–6). In this study, both low and very low food security were categorised as food insecurity. Missing values (*n* 6) were imputed following U.S. Department of Agriculture guidelines^(30)^. Briefly, the guidelines account for the sequential ordering of questions by severity, as individuals affirming an item typically affirm all less severe items, while those denying an item typically deny more severe items.

### Data analysis

All statistical analyses were conducted using R (R Core Team, 2024). All alpha levels were set to 0·05. Descriptive statistics (i.e. means, standard deviations, frequencies and proportions) were calculated for baseline demographic characteristics. Differences in baseline demographic characteristics between the Farm Share and control groups were assessed using the Wilcoxon rank sum test, Pearson’s *χ* ^2^ test and Fisher’s exact test. *P* values were corrected using false discovery rate correction for multiple testing^(31)^.

The Farm Share effect on food security was assessed using generalised estimating equations with an exchangeable working correlation structure and a logit link^(32)^. Generalised estimating equations was selected over logistic mixed-effects models due to convergence issues and better model fit.^(33)^. The Farm Share effect on skin carotenoids, HEI and FV intake was assessed using mixed-effects linear regression models^(34)^. All models included random effects for individuals and fixed effects for time, treatment group and their interaction. Race and ethnicity were not included as covariates due to collinearity with birthplace (U.S.-born or foreign-born) as tested by variance inflation factor. All adjusted models included age, sex and birthplace as covariates, with smoking status additionally included in the skin carotenoid models due to their strong inverse correlation in previous literature^(25)^.

Due to reasons not explained by participants, a small number (*n* 8 out of 33) of Farm Share participants ended up not signing up for any Farm Share boxes but did complete baseline and follow-up assessments. Following the intent-to-treat principle, these participants were included in the primary analyses. A separate sensitivity analysis excluding these participants was also conducted.

## Results

### Baseline characteristics

A total of 123 participants were enrolled at baseline (Farm Share *n* 54, control *n* 69). The total sample was predominantly Black or African American, of Hispanic/Latino origin, foreign-born, single, female and had a high school education or lower. More than three-quarters (81·3 %) of the participants who chose ‘other’ race indicated a Hispanic/Latino origin in the text field. The Farm Share group had more participants identified as Black (*P* = 0·037), not Hispanic/Latino (*P* = 0·031) and U.S.-born (*P* < 0·001) compared with the control group. The majority of both the Farm Share (83·0 %) and control participants (70·6 %) were food insecure at baseline. The control group had significantly higher (*P* < 0·001) skin carotenoid scores than the Farm Share group at baseline. A summary of the baseline characteristics is in Table 1.

**Table 1.** Baseline characteristics of the study cohort Table 1 long description.

Variables	*n*	All		Farm Share^*^		Control^*^ pantry		*P* ^†^
		(*n* 123)		(*n* 54)^*^		(*n* 69)^*^		
		*n*	%	*n*	%	*n*	%	
Age, years								
Mean	123	55·3		56·3		55·3		0·999
sd		15·4		14·1		16·5		
Sex	123							0·389
Male		27	22·0 %	9	16·7 %	18	26·1 %	
Female		96	78·1 %	45	83·3 %	51	73·9 %	
Education	116							0·476
Less than a high school		49	42·2 %	21	40·4 %	28	43·8 %	
High school degree or GED		30	25·9 %	11	21·2 %	19	29·7 %	
Some college		20	17·2 %	9	17·3 %	11	17·2 %	
4-year college degree or higher		17	14·7 %	11	21·1 %	6	9·4 %	
Marital status	121							0·17
Single								
		85	70·3 %	43	79·6 %	42	62·7 %	
Married		28	23·1 %	10	18·5 %	18	26·9 %	
Unmarried but living with a partner		8	6·6 %	1	1·9 %	7	10·5 %	
Race^‡^	123							
Native American /Alaska Native		5	4·1 %	4	7·4 %	1	1·5 %	0·351
Asian		1	0·81 %	1	1·9 %	0	0	0·597
Black or African American		25	20·3 %	17	31·5 %	8	11·6 %	0·037
Native Hawaiian/Other Pacific Islander		0	0	0	0	0	0	–
White or Caucasian		22	17·9 %	10	18·5 %	12	17·4 %	0·927
Other^§^		69	56·1 %	23	42·6 %	46	66·7 %	0·037
Ethnicity^‡^	123							
Not Hispanic/Latino		28	22·7 %	19	35·2 %	9	13·4 %	0·031
Mexican, Mexican American, Chicano		12	9·8 %	7	13·0 %	5	7·3 %	0·468
Puerto Rican		13	10·6 %	8	14·8 %	5	7·3 %	0·351
Cuban		1	0·81 %	0	0	1	1·5 %	0·999
Other Hispanic/Latino		61	49·6 %	20	37·0 %	41	59·4 %	0·058
Foreign-born	123	91	74·0 %	30	56·6 %	61	88·4 %	<0·001
Weekly work hours	114							0·55
Not working		78	68·4 %	38	74·5 %	40	63·5 %	
Less than 30 h		22	19·3 %	8	15·7 %	14	22·2 %	
Between 30 and 40 h		11	9·7 %	3	5·9 %	8	12·7 %	
More than 40 h		3	2·6 %	2	3·9 %	1	1·6 %	
Household annual income	114							0·413
< $20 000		95	83·3 %	45	86·6 %	50	80·7 %	
$20 000–$50 000		15	13·2 %	7	13·5 %	8	12·9 %	
> $50 000		4	3·5 %	0	0	4	6·5 %	
SNAP or WIC recipient	121	63	52·1 %	33	63·5 %	30	43·5 %	0·091
Household number								
Mean	113	2·9		2·6		3·2		0·17
sd		1·8		1·68		1·86		
Self-rated health	123							0·754
Excellent		15	12·2 %	9	16·7 %	6	8·7 %	
Good		51	41·5 %	21	38·9 %	30	43·5 %	
Fair		42	34·2 %	17	31·5 %	25	36·2 %	
Poor		15	12·2 %	7	13·0 %	8	11·6 %	
BMI, kg/m^2^	123							
Mean		27·7		27·6		27·8		0·927
sd		5·7		6·1 %		5·3 %		
Food security status	121							0·254
Food insecure		92	76·0 %	44	83·0 %	48	70·6 %	
Food secure		29	24·0 %	9	17·0 %	20	29·4 %	
		Mean	sd	Mean	sd	Mean	sd	
Skin carotenoids (Veggie Meter score)	123	314	112·3	264	99·4	353	106·9	<0·001
Daily FV intake, cup equivalents	120	2·3	0·90	2·4	1·06	2·3	0·76	0·927
HEI total score	88	62·1	11·67	60·6	10·6	63·2	12·4	0·389

SNAP, Supplemental Nutrition Assistance Program; WIC, Special Supplemental Nutrition Program for Women, Infants, and Children; FV, fruits and vegetables; HEI, healthy eating index.

* Mean (sd); *n* (%).

† Wilcoxon rank sum test; Pearson’s *χ* ^2^ test; Fisher’s exact test. False discovery rate correction for multiple testing.

‡ Sum is greater than the total sample size because multiple options were selected by individuals with multiple racial and ethnic identities.

§ Most individuals (81·3 %) who selected ‘other’ indicated Hispanic/Latino.

### Data collection fidelity

The retention rate at 3 months was 65·0 % (Farm Share *n* 33, control *n* 47). Reasons for loss to follow-up included no response to calls and texts, disconnect or out-of-service phone numbers, sickness and life events (e.g. family passing or becoming ill). There were no significant differences in baseline demographic and outcome variables between those with and without follow-up assessments (see online supplementary material, Supplemental Table 1S). Several participants (*n* 10) were not able to complete in-person assessments (i.e. Veggie Meter®) at follow-up due to mobility issues or life events and completed the survey via phone. Most Farm Share participants (*n* 25) picked up produce boxes at least once during the study period, with an average pick-up frequency of 2·84 times, ranging from 1 to 7 times. Of these, 18 participants (58·1 %) picked up less than once per month, while thirteen participants (41·9 %) picked up more than once per month. Over the 3-month study period, participants had a maximum of 6–7 pickup opportunities available, with slight variation due to scheduling accommodations for follow-up assessments. At baseline, nearly a quarter (71·5 %) of participants completed at least one diet recall, and fewer (60·2 %) completed both diet recalls. At follow-up, sixty-six participants completed at least one diet recall, and fifty-nine participants completed both diet recalls. The complete diet recall completion rates are in online supplementary material, Supplemental Table 2S.

### Program outcomes

**Figure 1.**
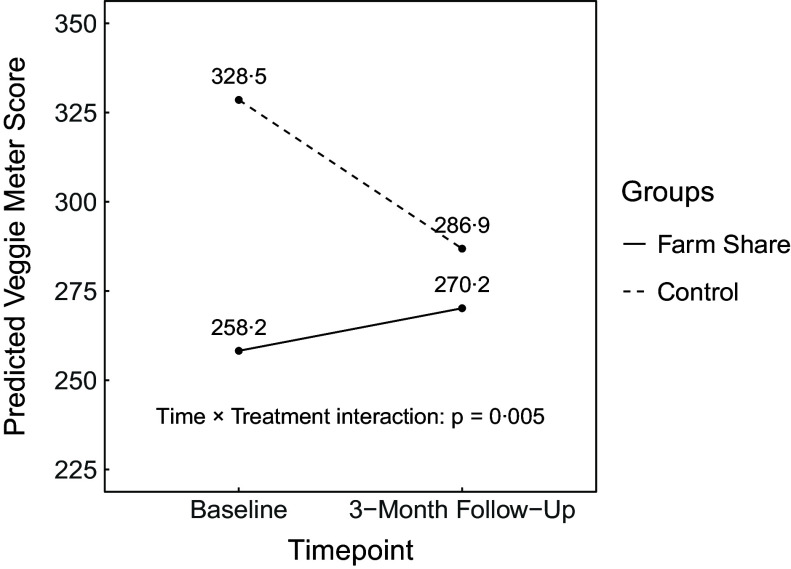
Changes in skin carotenoid scores between Farm Share (*n* 33) and control (*n* 47) groups over 3 months, adjusted for age, sex, birthplace and smoking status.

The changes in outcome variables at the 3-month follow-up are summarised in Table 2. The regression statistics are presented in online supplementary material, Supplemental Table 3S. The skin carotenoid scores significantly improved in the Farm Share group both unadjusted model (*P* = 0·002) and model adjusted for covariates such as smoking status, age, sex and birthplace (*P* = 0·005). While the control group had higher skin carotenoid scores at baseline, the score declined by follow-up, whereas the Farm Share group demonstrated an increase (Figure 1).

**Table 2.** Change in outcome variables at 3-month follow-up from unadjusted and adjusted regression models^*^

Variables	Groups	Baseline	Follow-up	Change	*P* ^†^
Unadjusted					
Food security (probability)	Farm Share	0·17	0·30	+0·13	0·162
	Control	0·29	0·26	−0·03	
Veggie Meter score	Farm Share	264	289	+25	0·002
	Control	353	311	−42	
Daily FV intake (cups)	Farm Share	2·39	2·54	+0·15	0·250
	Control	2·27	2·24	−0·03	
HEI total score	Farm Share	61·0	55·0	−6	0·033
	Control	63·3	63·6	−0·3	
Adjusted					
Food security (probability)	Farm Share	0·10	0·19	+0·09	0·187
	Control	0·33	0·27	−0·06	
Veggie Meter score	Farm Share	258	270	+12	0·005
	Control	329	287	−42	
Daily FV intake (cups)	Farm Share	2·51	2·69	+0·18	0·184
	Control	2·38	2·35	−0·03	
HEI total score	Farm Share	58·4	52·1	−6·3	0·031
	Control	59·5	59·6	+0·1	

FV, fruits and vegetables; HEI, healthy eating index.

* Estimated probability (food security) and means (all other variables) from generalised estimating equations and mixed-effects linear regression models. Adjusted models included age, sex (female/male), birthplace (US-born/Foreign-born) and (in skin carotenoid model only) smoking status (Yes/No) as covariates.

† *P* represent the time × treatment interaction term from the regression models, which tests the difference-in-differences effect between Farm Share and control groups.

While the changes in estimated probability of being food secure and daily FV intake favored the Farm Share group, they were not significantly different between groups (*P* = 0·187 and *P* = 0·184, respectively).

HEI total scores declined in both groups, with a slightly larger decrease in the Farm Share group. Of the thirteen HEI sub-components, only saturated fats (*P* = 0·027) and fatty acids (*P* = 0·053) showed significant differences. Saturated fats and fatty acids HEI decreased in the Farm Share group (7·35–6·21 and 5·96–4·67, respectively) but increased in the control group (7·89–8·49 and 6·22–6·81), displaying opposite trends.

The results of the sensitivity analysis indicated that excluding Farm Share participants who did not sign up for produce boxes did not change the primary analysis outcomes (see online supplementary material, Supplemental Table 4S).

## Discussion

### Summary of main findings

The purpose of this pilot study was to assess the preliminary effectiveness of Farm Share, a food pantry-based, subsidised CSA program in a large metropolitan city in the USA, and to examine the feasibility of this study approach. Three-month Farm Share participation resulted in a significant increase in skin carotenoids compared with the control group. The study was overall feasible, though challenges with recruitment, retention and administering 24-h diet recalls were noted.

Aligned with the only two studies examining skin carotenoid levels among CSA participants^(16,18)^, this study observed an estimated twelve-point increase in skin carotenoid scores following 3 months of Farm Share participation. A pilot study evaluating a culturally tailored subsidised CSA for Chinese American communities reported a sixty-point increase following 20 weeks of weekly participation in Brooklyn, NY^(16)^. The larger effect size compared with our findings likely reflects the longer intervention duration and higher participant engagement, with 90 % purchasing weekly shares^(16)^. Similarly, F3HK, the multi-state randomised controlled trial found increased skin carotenoids after one growing season averaging 21 weeks of subsidised CSA, though direct effect size comparison was not possible due to different measurement methods using Raman resonance spectroscopy^(18)^. Notably, despite well-documented correlations between skin carotenoids and FV intake, the increase in skin carotenoids in our study was not accompanied by significant changes in self-reported FV intake^(35,36)^. This disparity may reflect differences in measurement approaches. The Dietary Screener Questionnaire evaluates dietary patterns across FV categories, while the Veggie Meter® captures skin carotenoid levels of lycopene, beta-carotene, alpha-carotene, beta-cryptoxanthin, zeaxanthin and lutein^(23)^. Consequently, individuals consuming FV low in carotenoids may exhibit relatively low skin carotenoid scores despite adequate intake^(37)^. The culturally tailored CSA in Brooklyn resulted in significant increases in produce consumption variety, but not quantity^(16)^. While strong correlations between FV intake and skin carotenoid scores are well documented^(35,36)^, future research could benefit from examining both the quantity and variety of FV to better understand this relationship in populations with low income. Additionally, participants enrolled across different seasons likely experienced varying types of seasonal produce, which could potentially influence dietary outcomes. While our concurrent enrollment of both study groups over the same timeframe may have partly mitigated seasonal effects on between-group comparisons, future CSA research would benefit from collecting data on produce box content to better understand how seasonal variation influences program effectiveness.

Diet quality denoted by HEI declined in the Farm Share group, particularly the Saturated fats and fatty acids HEI scores. These findings suggest that interventions focused on increasing FV intake may have unintended impacts on other dietary components and overall diet quality. HEI was assessed in two other CSA evaluation studies, only one yielded significant improvement^(17,18)^. HEI total score improved following a rural, health centre-based CSA in Massachusetts^(17)^. In comparison, the current study sample had lower income, less educational attainment, more individuals from racial and ethnic minority backgrounds and more foreign-born individuals. The demographic and geographic differences may relate to different food environment access and dietary habits^(38)^, potentially explaining the differential impact of CSA on HEI. Future research examining optimal subsidised CSA program designs that support overall diet quality improvement will be beneficial.

The long-term impact of subsidised CSA on food security calls for further investigation. While two clinic-based subsidised CSA programs (23 and 24 weeks) demonstrated significant post-intervention improvements in food security^(15,17)^, participant replacement due to high dropouts during one study may have influenced the results^(15)^. The F3HK study revealed that improved food security status achieved immediately post-CSA intervention was diminished by the following spring^(18)^, highlighting the need for sustained food access beyond the intervention period. Food security encompasses four dimensions: availability, accessibility, utilisation, and stability^(39)^. Subsidised CSA could support food availability and accessibility but may have limited impact on utilisation and stability, which are affected by numerous personal, social and environmental factors such as household composition and cultural beliefs^(40)^. While the inconsistent pick-up frequencies observed in our study might have reduced the likelihood of food stability, Farm Share automatically enrolled participants as food pantry clients, thereby providing continued access to food and social services independent of Farm Share pick-ups. Future research may benefit from examining pantry-based subsidised CSA’s longitudinal effects on food security and its effectiveness across settings through comparative trials.

### Study feasibility

While this study design was overall feasible, logistical constraints should be recognised. First, phone-based dietary recalls emerged as a key logistical challenge in our study design. Nonresponsive participants and out-of-service phones led to missing data, barriers previously documented in food pantry populations^(41)^. Additionally, even when participants could be reached within 1 week of the collection point, many required rescheduling due to unavailability, resulting in data collection delays. These communication difficulties made it impractical to conduct recalls on both weekdays and weekends, potentially limiting data representativeness^(42)^. Future studies with this population could benefit from implementing multiple communication channels (e.g. phone, text and in-person contact), enhancing participant incentives and increasing research staff capacity. Alternatively, brief dietary questionnaires might be more feasible in this setting.

Furthermore, identifying a suitable control group presented unexpected challenges. Despite email and phone outreach, no contacted food pantries responded to our inquiries. While the eventual control pantry matched the CSA pantry in size and reach, demographic differences existed between service areas. Additionally, though the control pantry did not offer a CSA program, it provided fresh produce through regular services. Selecting a pantry with minimal fresh produce could have demonstrated a more pronounced intervention effect but would likely have indicated limited organisational capacity, potentially compromising research collaboration feasibility. Allocating sufficient time for control site identification and developing strategies to support partnerships with resource-limited community sites may benefit future research.

Finally, several recruitment and operational challenges limited the study sample size. The CSA season timing (March to November) constrained our enrollment period to March through August to ensure participants had at least 3 months of Farm Share exposure. Our pilot study operated with limited resources, including one part-time research assistant coordinating data collection across both pantries. Recruitment and data collection schedules were dependent on pantry operational logistics, including availability of pantry representatives who assisted with recruitment as volunteers beyond their full-time responsibilities. Study activities were periodically paused when pantries hosted other organisations or when key staff were unavailable. Our sample size was further impacted by participant attrition, primarily due to discontinued phone service and personal health issues, which are common challenges in programs for populations affected by poverty^(43,44)^. In our study, pantry staff played a crucial role in reaching with some participants with disconnected phones. These coordination challenges highlight the need for increased staffing investment in both research teams and partner organisations for future trials. Additionally, future studies would benefit from enhanced recruitment strategies, such as expanded outreach during pre-season months (January–February) when potential participants may be more receptive to program information. Lastly, a multi-year, multi-cohort design may be necessary to achieve adequate sample sizes for fully powered trials in this setting.

### Limitations

The pilot study makes a unique contribution to the subsidized CSA literature by focusing on an innovative CSA setting and an understudied population. Nonetheless, several limitations warrant consideration. While hypothesis testing was not the primary aim, the small sample size could have potentially affected the reliability of significant findings in skin carotenoids. Hence, these findings warrant replication in fully powered trials.

Selection bias presents another limitation, as CSA participants typically demonstrate more positive FV knowledge, attitude and beliefs and higher intake compared with their counterparts^(45)^. Our sample showed slightly higher baseline values for FV intake, skin carotenoid scores and overall HEI compared with food pantry clients in other studies^(46,47)^, though these baseline values were not different between Farm Share and control groups, with the exception of skin carotenoids. We acknowledge that regression to the mean may partially explain the differential changes in skin carotenoids between groups, given the baseline differences. While the inclusion of a control group and significant interaction effects suggest an intervention effect beyond regression to the mean alone, our nonrandomised design limits our ability to fully control for baseline differences. Additionally, Farm Share participants likely possessed unmeasured characteristics such as adequate kitchen facilities, cooking space, housing stability and preference for FV that may differ from the general food pantry population. Farm Share participants may have displayed a constellation of unmeasured psychological, behavioural and structural factors that made them amenable to participating in a subsidized CSA intervention. Understanding the characteristics of individuals who opt into such programs *v*. those who decline remains a research gap. However, we were unable to collect data from food pantry clients who were offered Farm Share but chose not to enroll, limiting our ability to identify potential baseline ‘tipping points’ for programme participation.

The considerable variation in pickup frequencies represents an important limitation. Pickup challenges related to work schedules, transportation barriers, forgetfulness and health issues have been previously documented in subsidised CSA research^(13)^. Factors associated with higher CSA engagement in the F3HK study included living in micropolitan rather than rural areas, households with multiple adults, annual income above $25 000, college education, married status and availability of multiple share size options^(48)^. While F3HK reported that 5 % of participants never collected CSA shares^(49)^, our study had a substantially higher rate of 24 % non-pickup among Farm Share participants. This low engagement pattern, combined with infrequent pickup among participating members, may undermine confidence in attributing observed outcomes to the intervention due to low exposure to Farm Share. Although sensitivity analyses excluding non-participants did not alter our findings, the overall low pickup frequency raises questions about CSA preferences and barriers specific to food pantry populations. While our pilot study design limited our ability to investigate these factors, future research could examine determinants of engagement to optimise subsidized CSA design and improve programme fidelity in food pantry settings.

External validity limitations stem from the unique characteristics of our study settings and population. The study pantries were among NYC’s largest food pantries, serving predominantly Spanish-speaking Latino and foreign-born clients in the most populous city in the U.S. These features may limit generalizability to rural or smaller-scale pantries. Additionally, the study timing coincided with the end of temporary COVID-19 U.S. governmental food assistance (SNAP) benefit increases in February 2023^(50)^. With nearly half of participants receiving SNAP or WIC, this reduction in benefits could have potentially influenced purchasing power and dietary behaviours. Despite these limitations, the study achieved its primary objective of assessing study feasibility.

### Future directions

The findings from this pilot study warrant further investigation of Farm Share with an adequately powered sample to evaluate effectiveness in improving dietary behaviours and food security. A larger trial would also enable dose–response analysis to investigate whether biweekly pickup is sufficient for achieving intended outcomes on dietary behaviours, identify characteristics that distinguish frequent *v*. infrequent participants and determine which participants would benefit from more or less frequent pickup schedules. To assess broader implementation potential, future research could examine cost-effectiveness of subsidised CSA programs in food pantry settings, particularly compared with other fresh produce programs in this setting. Qualitative methods would be valuable for assessing CSA preferences and participation barriers, especially among those who were offered the programme but declined to participate. This approach could help identify characteristics that differentiate participants from non-participants, advancing our understanding of baseline factors salient for engagement in subsidised CSA.

### Conclusions

This pilot study suggests the feasibility of evaluating a subsidized CSA program in an urban food pantry setting. The findings indicate preliminary effectiveness in improving skin carotenoid levels after 3 months of participation. While larger trials are critical to confirm these findings, this pilot study contributes preliminary evidence warranting further investigation of integrating subsidised CSA programs within food pantries to promote FV intake among communities experiencing food insecurity.

## Supporting information

10.1017/S136898002610264X.sm001 Liu et al. supplementary material Liu et al. supplementary material

## Supplementary material

For supplementary material accompanying this paper visit https://doi.org/10.1017/S136898002610264X

## Table 1. Long description

The table presents baseline characteristics of participants in a study, divided into three groups: All (n 123), Farm Share (n 54), and Control Pantry (n 69). It includes variables such as age, sex, education, marital status, race, ethnicity, weekly work hours, household annual income, household number, self-rated health, BMI, food security status, skin carotenoids, fruit and vegetable intake, and HEI total score. Each variable is presented with the number of participants (n) and percentage (%). Notable trends include the predominance of female participants, those with a high school education or lower, and those who are single. The majority of participants are Black or African American and of Hispanic/Latino origin. The Farm Share group had more participants identified as Black, not Hispanic/Latino, and U.S.-born compared with the control group. The majority of both groups were food insecure at baseline. The control group had significantly higher skin carotenoid scores than the Farm Share group.

Navigate back to Table 1..
