# Supplementary material for: Preliminary effectiveness of a food pantry-led community-supported agriculture program in the USA: a quasi-experimental pilot study
Source: Public Health Nutr. 2026 Jun 2;29(1):e130. doi: 10.1017/S136898002610264X (PMC13434551; doi:10.1017/S136898002610264X)
Supplement: Supplementary file 1 [file S136898002610264Xsup001.docx]

**Table 1S.** Baseline characteristics of participants with and without follow-up assessments.

| **Variables** | **N** | **With follow up**  **(n = 80)^1^** | **No follow-up**  **(n = 43)^1^** | **P values^2^** |
| --- | --- | --- | --- | --- |
| Site/group  Farm Share  Control | 123 | 33 (41.3%)  47 (58.8%) | 21 (48.8%)  22 (51.2%) | 0.762 |
| Age, y | 123 | 58.0 (13.6) | 50.3 (17.3) | 0.161 |
| Sex  Male  Female | 123 | 14 (17.5%)  66 (82.5%) | 13 (30.2%)  30 (70.0%) | 0.605 |
| Education  Less than a high school  High school degree or GED  Some college  4-year college degree or higher | 116 | 32 (42.1%)  20 (26.3%)  12 (15.8%)  12 (15.8%) | 17 (42.5%)  10 (25.0%)  8 (20.0%)  5 (12.5%) | 0.992 |
| Marital status  Single  Married  Unmarried but living with a partner | 121 | 51 (65.4%)  22 (28.2%)  5 (6.4%) | 34 (79.1%)  6 (14.0%)  3 (7.0%) | 0.747 |
| Race^3^  Native American /Alaska Native  Asian  Black or African American  Native Hawaiian/Other Pacific Islander  White or Caucasian  Other^4^ | 123 | 2 (2.5%)  1 (1.3%)  18 (22.5%)  0 (0)  12 (15.0%)  47 (58.8%) | 3 (7.0%)  0 (0)  7 (16.3%)  0 (0)  10 (23.3%)  22 (51.2%) | 0.762  0.999  0.762  NA  0.762  0.762 |
| Ethnicity^3^  Not Hispanic/Latino  Mexican, Mexican American, Chicano  Puerto Rican  Cuban  Other Hispanic/Latino | 123 | 18 (22.5%)  10 (12.5%)  8 (10.0%)  0 (0)  38 (47.5%) | 10 (23.3%)  2 (4.7%)  5 (11.6%)  1 (2.3%)  23 (53.5%) | 0.992  0.747  0.959  0.762  0.801 |
| Foreign-born | 123 | 59 (73.8%) | 32 (74.4%) | 0.992 |
| Weekly work hours  Not working  Less than 30 hours  Between 30-40 hours  More than 40 hours | 114 | 49 (65.3%)  17 (22.7%)  8 (10.7%)  1 (1.3%) | 29 (74.4%)  5 (12.8%)  3 (7.7%)  2 (5.1%) | 0.762 |
| Household annual income  < $20,000  $20,000 - $50,000  > $50,000 | 114 | 60 (79.0%)  13 (17.1%)  3 (4.0%) | 35 (92.1%)  2 (5.3%)  1 (2.6%) | 0.747 |
| SNAP or WIC recipient | 121 | 40 (50.6%) | 23 (54.8%) | 0.931 |
| Household number | 113 | 2.9 (1.7) | 3.0 (2.0) | 0.990 |
| Self-rated health  Excellent  Good  Fair  Poor | 123 | 8 (10.0%)  32 (40.0%)  28 (35.0%)  12 (15.0%) | 7 (16.3%)  19 (44.2%)  14 (32.6%)  3 (7.0%) | 0.778 |
| Body mass index, kg/m^2^ | 123 | 28.4 (5.1) | 26.3 (6.5) | 0.161 |
| Food security status  Food insecure  Food secure | 121 | 58 (73.4%)  21 (26.6%) | 34 (81.0%)  8 (19.1%) | 0.762 |
| Veggie Meter score | 123 | 332 (112.5) | 280 (105.3) | 0.161 |
| Daily FV intake, cup equivalents | 120 | 2.31 (0.76) | 2.34 (1.1) | 0.762 |
| HEI total score | 88 | 61.9 (11.9) | 62.6 (11.1) | 0.937 |

SNAP, Supplemental Nutrition Assistance Program; WIC, Special Supplemental Nutrition Program for Women, Infants, and Children; FV, fruits and vegetables; HEI, Healthy Eating Index.

^1^ Mean (SD); N (%)

^2^ Wilcoxon rank sum test; Pearson’s Chi-squared test; Fisher’s exact test. False discovery rate correction for multiple testing.

^3^ Sum is greater than the total sample size because multiple options were selected by individuals with multiple racial and ethnic identities.

**Table 2S.** Diet recall completion rate at baseline and follow-up.

|  |  | **Total** | **Farm Share** | **Control** |
| --- | --- | --- | --- | --- |
| Baseline | One recall | 88 | 38 | 50 |
|  | Two recalls | 74 | 30 | 44 |
| Follow-up | One recall | 66 | 31 | 35 |
|  | Two recalls | 59 | 26 | 33 |
| At least one recall at both timepoints | | 58 | 27 | 31 |
| All four recalls | | 45 | 20 | 25 |

**Table 3S.** Results of regression analysis on primary outcomes.^1^

**1. Food insecurity**

| **Characteristic** | $\boldsymbol{\beta}\mathbf{̂}$ | **Std. Error** | **Wald test statistics** | **P values** |
| --- | --- | --- | --- | --- |
| **Unadjusted model** |  |  |  |  |
| Intercept | -1.59 | 0.37 | 18.82 | <0.001 |
| Follow-up - baseline (timepoint) | 0.75 | 0.53 | 2.05 | 0.152 |
| Control - intervention (site) | 0.71 | 0.45 | 2.47 | 0.116 |
| Timepoint x site | -0.95 | 0.68 | 1.96 | 0.162 |
|  |  |  |  |  |
| **Adjusted model** |  |  |  |  |
| Intercept | -3.47 | 1.18 | 8.63 | 0.003 |
| Follow-up - baseline (timepoint) | 0.70 | 0.60 | 1.36 | 0.244 |
| Control - intervention (site) | 1.45 | 0.59 | 6.12 | 0.013 |
| Age | 0.03 | 0.01 | 4.90 | 0.068 |
| Female – Male (sex) | 1.25 | 0.49 | 6.65 | 0.010 |
| Foreign – U.S. (birthplace) | -1.45 | 0.48 | 9.31 | 0.002 |
| Timepoint x site | -0.97 | 0.73 | 1.74 | 0.187 |

**2. Skin carotenoids (Veggie Meter scores)**

| **Characteristic** | $\boldsymbol{\beta}\mathbf{̂}$ | **Std. Error** | **Df** | **t-value** | **P values** |
| --- | --- | --- | --- | --- | --- |
| **Unadjusted model** |  |  |  |  |  |
| Intercept | 263.9 | 13.8 | 143.9 | 19.08 | <0.001 |
| Follow-up - baseline (timepoint) | 15.7 | 13.9 | 78.9 | 1.13 | 0.263 |
| Control - intervention (site) | 88.8 | 18.5 | 143.9 | 4.81 | < 0.001 |
| Timepoint x site | -57.1 | 18.2 | 78.4 | -3.13 | 0.002 |
|  |  |  |  |  |  |
| **Adjusted model** |  |  |  |  |  |
| Intercept | 131.2 | 61.9 | 140.2 | 2.12 | 0.036 |
| Follow-up - baseline (timepoint) | 11.9 | 14.0 | 77.9 | 0.85 | 0.399 |
| Control - intervention (site) | 70.3 | 19.3 | 139.4 | 3.65 | < 0.001 |
| Smoker – non-smoker | -15.9 | 26.3 | 170.7 | -0.60 | 0.546 |
| Age | 1.06 | 0.56 | 123.4 | 1.90 | 0.060 |
| Female – Male (sex) | -1.71 | 22.8 | 129.9 | -0.07 | 0.940 |
| Foreign – U.S. (birthplace) | 51.69 | 21.8 | 137.3 | 2.37 | 0.019 |
| Timepoint x site | -53.6 | 18.4 | 78.4 | -2.91 | 0.005 |

**3. FV intake**

| **Characteristic** | $\boldsymbol{\beta}\mathbf{̂}$ | **Std. Error** | **Df** | **t-value** | **P values** |
| --- | --- | --- | --- | --- | --- |
| **Unadjusted model** |  |  |  |  |  |
| Intercept | 2.39 | 0.12 | 140.2 | 20.23 | <0.001 |
| Follow-up - baseline (timepoint) | 0.15 | 0.12 | 77.3 | 1.27 | 0.208 |
| Control - intervention (site) | -0.12 | 0.16 | 140.9 | -0.74 | 0.459 |
| Timepoint x site | -0.18 | 0.16 | 76.6 | -1.15 | 0.250 |
|  |  |  |  |  |  |
| **Adjusted model** |  |  |  |  |  |
| Intercept | 3.24 | 0.46 | 124.6 | 7.04 | <0.001 |
| Follow-up - baseline (timepoint) | 0.17 | 0.12 | 77.3 | 1.47 | 0.147 |
| Control - intervention (site) | -0.13 | 0.17 | 135.8 | -0.74 | 0.460 |
| Age | -0.0014 | 0.0047 | 142.8 | -0.30 | 0.768 |
| Female – Male (sex) | -0.37 | 0.19 | 113.0 | -2.00 | 0.048 |
| Foreign – U.S. (birthplace) | -0.063 | 0.18 | 120.6 | -0.35 | 0.723 |
| Timepoint x site | -0.21 | 0.16 | 76.6 | -1.34 | 0.184 |

**4. Total HEI**

| **Characteristic** | $\boldsymbol{\beta}\mathbf{̂}$ | **Std. Error** | **Df** | **t-value** | **P values** |
| --- | --- | --- | --- | --- | --- |
| **Unadjusted model** |  |  |  |  |  |
| Intercept | 70.0 | 1.92 | 134.4 | 31.7 | <0.001 |
| Follow-up - baseline (timepoint) | -5.95 | 2.14 | 72.0 | -2.79 | 0.007 |
| Control - intervention (site) | 2.32 | 2.55 | 133.7 | 0.91 | 0.365 |
| Timepoint x site | 6.29 | 2.91 | 73.3 | 2.16 | 0.033 |
|  |  |  |  |  |  |
| **Adjusted model** |  |  |  |  |  |
| Intercept | 27.7 | 8.00 | 102.7 | 3.47 | <0.001 |
| Follow-up - baseline (timepoint) | -6.27 | 2.15 | 74.4 | -2.92 | 0.005 |
| Control - intervention (site) | 1.14 | 2.59 | 130.1 | 0.44 | 0.661 |
| Age | 0.25 | 0.075 | 91.9 | 3.30 | 0.001 |
| Female – Male (sex) | 4.39 | 2.89 | 99.9 | 1.52 | 0.132 |
| Foreign – U.S. (birthplace) | 6.63 | 2.65 | 98.7 | 2.50 | 0.014 |
| Timepoint x site | 6.39 | 2.92 | 75.9 | 2.19 | 0.031 |

FV, fruits and vegetables; HEI, Healthy Eating Index; U.S., the United States (of America).

^1^ Results from generalized estimating equations (food insecurity) and mixed-effect linear regression models (skin carotenoids, FV intake, and total HEI). Adjusted models included age, sex (Female/Male), birthplace (US-born/Foreign-born), and (in skin carotenoid model only) smoking status (Yes/No) as covariates.

**Table 4S.** Sensitivity analysis of changes in primary outcomes excluding Farm Share participants who did not receive CSA^1^

**1. Food insecurity**

| **Characteristic** | $\boldsymbol{\betâ}$ | **Std. Error** | **Wald test statistics** | **P values** |
| --- | --- | --- | --- | --- |
| **Unadjusted model** |  |  |  |  |
| Intercept | -1.59 | 0.37 | 18.8 | <0.001 |
| Follow-up - baseline (timepoint) | 0.72 | 0.56 | 1.67 | 0.196 |
| Control - intervention (site) | 0.71 | 0.45 | 2.47 | 0.116 |
| Timepoint x site | -0.92 | 0.70 | 1.70 | 0.192 |
|  |  |  |  |  |
| **Adjusted model** |  |  |  |  |
| Intercept | -3.60 | 1.19 | 9.11 | 0.003 |
| Follow-up - baseline (timepoint) | 0.60 | 0.63 | 0.91 | 0.340 |
| Control - intervention (site) | 1.37 | 0.57 | 5.66 | 0.017 |
| Age | 0.03 | 0.01 | 4.60 | 0.032 |
| Female – Male (sex) | 1.20 | 0.48 | 6.25 | 0.012 |
| Foreign – U.S. (birthplace) | -1.29 | 0.49 | 6.98 | 0.008 |
| Timepoint x site | -0.86 | 0.76 | 1.30 | 0.254 |

**2. Skin carotenoids (Veggie Meter scores)**

| **Characteristic** | $\boldsymbol{\betâ}$ | **Std. Error** | **Df** | **t-value** | **P values** |
| --- | --- | --- | --- | --- | --- |
| **Unadjusted model** |  |  |  |  |  |
| Intercept | 263.9 | 13.9 | 143.3 | 19.02 | <0.001 |
| Follow-up - baseline (timepoint) | 18.2 | 15.5 | 76.4 | 1.18 | 0.243 |
| Control - intervention (site) | 88.8 | 18.5 | 143.3 | 4.80 | <0.001 |
| Timepoint x site | -59.4 | 19.6 | 75.0 | -3.04 | 0.003 |
|  |  |  |  |  |  |
| **Adjusted model** |  |  |  |  |  |
| Intercept | 131.5 | 62.3 | 139.8 | 2.11 | 0.037 |
| Follow-up - baseline (timepoint) | 14.2 | 15.6 | 75.9 | 0.91 | 0.366 |
| Control - intervention (site) | 70.6 | 19.3 | 139.0 | 3.65 | <0.001 |
| Smoker – non-smoker | -17.3 | 26.7 | 167.6 | -0.66 | 0.513 |
| Age | 1.11 | 0.56 | 124.3 | 1.98 | 0.05003 |
| Female – Male (sex) | -2.06 | 22.89 | 128.7 | -0.09 | 0.929 |
| Foreign – U.S. (birthplace) | 50.2 | 22.01 | 138.1 | 2.28 | 0.024 |
| Timepoint x site | -55.8 | 19.7 | 75.4 | -2.82 | 0.006 |

**3. FV intake**

| **Characteristic** | $\boldsymbol{\betâ}$ | **Std. Error** | **Df** | **t-value** | **P values** |
| --- | --- | --- | --- | --- | --- |
| **Unadjusted model** |  |  |  |  |  |
| Intercept | 2.39 | 0.118 | 139.4 | 20.26 | <0.001 |
| Follow-up - baseline (timepoint) | 0.15 | 0.13 | 75.1 | 1.12 | 0.270 |
| Control - intervention (site) | -0.12 | 0.16 | 140.2 | -0.74 | 0.458 |
| Timepoint x site | -0.17 | 0.17 | 73.4 | -1.05 | 0.295 |
|  |  |  |  |  |  |
| **Adjusted model** |  |  |  |  |  |
| Intercept | 3.27 | 0.46 | 124.6 | 7.12 | <0.001 |
| Follow-up - baseline (timepoint) | 0.17 | 0.13 | 75.2 | 1.31 | 0.194 |
| Control - intervention (site) | -0.12 | 0.17 | 135.4 | -0.74 | 0.463 |
| Age | -0.002 | 0.005 | 144.5 | -0.42 | 0.673 |
| Female – Male (sex) | -0.37 | -0.18 | 112.2 | -1.99 | 0.049 |
| Foreign – U.S. (birthplace) | -0.067 | 0.18 | 122.5 | -0.37 | 0.712 |
| Timepoint x site | -0.21 | 0.17 | 73.6 | -1.24 | 0.219 |

**4. Total HEI**

| **Characteristic** | $\boldsymbol{\betâ}$ | **Std. Error** | **Df** | **t-value** | **P values** |
| --- | --- | --- | --- | --- | --- |
| **Unadjusted model** |  |  |  |  |  |
| Intercept | 60.9 | 1.95 | 127.7 | 31.2 | <0.001 |
| Follow-up - baseline (timepoint) | -6.59 | 2.32 | 70.0 | -2.84 | 0.006 |
| Control - intervention (site) | 2.39 | 2.59 | 127.8 | 0.92 | 0.358 |
| Timepoint x site | 6.91 | 3.04 | 69.2 | 2.27 | 0.026 |
|  |  |  |  |  |  |
| **Adjusted model** |  |  |  |  |  |
| Intercept | 27.7 | 8.22 | 101.4 | 3.37 | 0.001 |
| Follow-up - baseline (timepoint) | -6.98 | 2.32 | 73.8 | -3.01 | 0.004 |
| Control - intervention (site) | 1.25 | 2.64 | 124.1 | 0.47 | 0.638 |
| Age | 0.26 | 0.08 | 94.7 | 3.31 | 0.001 |
| Female – Male (sex) | 4.34 | 2.95 | 96.8 | 1.47 | 0.145 |
| Foreign – U.S. (birthplace) | 6.34 | 2.76 | 101.1 | 2.30 | 0.024 |
| Timepoint x site | 7.08 | 3.04 | 73.0 | 2.33 | 0.023 |

FV, fruits and vegetables; HEI, Healthy Eating Index; U.S., the United States (of America).

^1^ Results from generalized estimating equations (food insecurity) and mixed-effect linear regression models (skin carotenoids, FV intake, and total HEI). Adjusted models included age, sex (Female/Male), birthplace (US-born/Foreign-born), and (in skin carotenoid model only) smoking status (Yes/No) as covariates.
